# Glycogen synthase kinase 3 (GSK-3) controls T-cell motility and interactions with antigen presenting cells

**DOI:** 10.1186/s13104-020-04971-0

**Published:** 2020-03-18

**Authors:** Alison Taylor, Christopher E. Rudd

**Affiliations:** 1 Leeds Institute of Medical Research, School of Medicine, University of Leeds, Wellcome Trust Brenner Building, St James’s University Hospital, Leeds, LS9 7TF UK; 2 Cell Signalling Section, Department of Pathology, University of Cambridge, Tennis Court Road, Cambridge, CB2 1Q UK; 3 Division of Immunology-Oncology Research Center, Maisonneuve-Rosemont Hospital, Montreal, QC H1T 2M4 Canada; 4 Département de Medicine, Université de Montréal, Montreal, QC H3C 3J7 Canada

**Keywords:** T-cells, GSK-3, Motility, Cell contacts

## Abstract

**Objective:**

The threonine/serine kinase glycogen synthase kinase 3 (GSK-3) targets multiple substrates in T-cells, regulating the expression of Tbet and PD-1 on T-cells. However, it has been unclear whether GSK-3 can affect the motility of T-cells and their interactions with antigen presenting cells.

**Results:**

Here, we show that GSK-3 controls T-cell motility and interactions with other cells. Inhibition of GSK-3, using structurally distinct inhibitors, reduced T-cell motility in terms of distance and displacement. While SB415286 reduced the number of cell-cell contacts, the dwell times of cells that established contacts with other cells did not differ for T-cells treated with SB415286. Further, the increase in cytolytic T-cell (CTL) function in killing tumor targets was not affected by the inhibition of motility. This data shows that the inhibition of GSK-3 has differential effects on T-cell motility and CTL function where the negative effects on cell–cell interactions is overridden by the increased cytolytic potential of CTLs.

## Introduction

T-cells are activated via a tyrosine kinase phosphorylation cascade that is initiated when the T-cell receptor (TCR) recognises foreign antigens, or tumor neoantigens, as presented by major histocompatibility (MHC) antigens. The cascade is initiated by the immune cell *src* kinase p56^lck^ which we showed binds to the cytoplasmic tails of co-receptors CD4 and CD8 [1–3]. Co-recognition of MHC-antigen by the TCR, and CD4 or CD8, brings p56^lck^ into proximity of the TCR for the phosphorylation of immunoreceptor tyrosine-based activation motifs (ITAMs) in the cytoplasmic tails of the CD3 and the ζ-subunits of the TCR-CD3 complex [2]. Phospho-ITAMs then bind to a second tyrosine kinase, zeta-chain associated protein kinase 70 (ZAP-70) which is further activated by p56^lck^ [4]. p56^lck^ and ZAP-70 phosphorylate downstream substrates that include adaptors or scaffolds which form multimeric complexes that integrate signals for T-cell effector functions. Examples of key adaptors include the linker for activation of T-cells (LAT) [5] and Src homology (SH)2 domain-containing leukocyte protein-76 (SLP-76) [6] which regulate intracellular calcium, or adhesion and degranulation-promoting adapter protein (ADAP) and Src kinase-associated phosphoprotein 1 (SKAP1) which activate LFA-1 adhesion [7–9].

By contrast, glycogen synthase kinase 3 (GSK-3) is a serine/threonine kinase that is active in resting T-cells and is inactivated upon T-cell activation [10, 11]. Isoforms of GSK-3 α and β differ in their N- and C-terminal sequences. TCR ligation induces GSK-3 inactivating phosphorylation [12–14], while the expression of active GSK-3β (GSK-3βA9) inhibits the proliferation of T-cells [12]. GSK-3 phosphorylation also regulates cellular metabolism [15] and microtubule-associated protein 2C (MAP2C) regulation of microtubule re-modelling [16, 17]. Protein kinase B (PKB/AKT) and its downstream target GSK-3 in T-cells appear to operate independently of guanine nucleotide exchange factor VAV-1 [13]. Further, in CD4^+^ T-cells, GSK-3 promotes the exit of nuclear factor of activated T-cells (NFAT) [18, 19]. Clinical trials using GSK-3 inhibitors have been undertaken in the treatment of type II diabetes and various neurological disorders [11, 20, 21]. Recently, we reported that the inactivation of GSK-3α/β specifically down-regulates PD-1 expression for enhanced cytolytic T-cell (CTL) function and the clearance of infection by Murid herpes virus-4 and lymphocytic choriomeningitis virus (LCMV) clone (Cl) 13 [22]. Further, we showed that GSK-3 inactivation is as effective as anti-PD-1 blockade in the regression of melanoma and lymphoma tumors [23, 24].

In this study, we assessed whether GSK-3 inhibition affects T-cell movement and interactions with other cells. Structurally distinct inhibitors of GSK-3 reduced T-cell motility as measured by velocity, distance and displacement. The consequence of this was to reduce the number of cell contacts with other cells. However, a concurrent increase in CTL function in killing tumor targets was not substantially affected by the inhibitory effect of GSK-3 inhibition on T-cell motility.

## Main text

### Methods

#### Mice and cells

Primary mouse T-cells (OT-1, C57BL/6, 6–8 weeks old) were isolated from spleens and cultured in vitro in RPMI 1640 medium supplemented with 10% FCS, 50 μM β-mercaptoethanol, 2 mM l-glutamine, 100 U/ml penicillin and streptomycin (GIBCO). Spleen cells were treated with a hypotonic buffer containing 0.15 M NH4CL, 10 mM KHCO3 and 0.1 mM EDTA, pH 7.2 to eliminate red blood cells before suspension in supplemented RPMI 1640 medium. A T-cell enriched population was purified by use of T-cell purification columns (R&D Systems, Minneapolis, MN). All mouse experiments were approved by the Home Office UK (PPL No. 70/7544). EL4 lymphoma cells were cultured in RPMI medium that was supplemented as above.

#### Cytotoxicity assays

OVA specific CD8^+^ CTLs were generated by incubating isolated splenocytes from OT-1 Tg mice with SIINFEKL peptide of OVA (OVA_257–264_) at 10 ng/mL for 5–7 days. For in vitro cytotoxic assays, T-cells were plated in 96-well plates at the start of culture with activating EL4 cells (EL4-OVA) pulsed with OVA_257–264_ peptide. EL4 cells were incubated with 10 nM OVA_257–264_ peptide (Bachem) for 1 h at 37 °C prior to co-culture at a ratio of 1:5 of EL4 and T-cell. CTLs were generated in the presence or absence of GSK-3 inhibitor for 7 days prior to co-culture. GSK-3 inhibitors SB415286, SB216763 (Abcam plc) and L803-mts (Tocris) were reconstituted in DMSO to give a stock solution of 25 mM and diluted to a concentration of 10µM in vitro. Cytotoxicity was assayed using a Cytotox 96 nonradioactive kit (Promega) following the instructions provided.

#### Live cell imaging

T-cells were labelled using Carboxyfluorescein succinimidyl ester (CFSE, Biolegend) and EL4-OVA target cells labelled with CellTracker^TM^ Red CMTPX Dye (Thermo Fisher Scientific). Imaging was performed using co-cultures on Poly-l-lysine-treated chambered glass culture slides (Lab-tek). Cells were imaged at the interface using a Zeiss LSM 510 confocal microscope using excitation wavelengths of 492 nm for CFSE and 577 nm for CellTracker^TM^ Red and a × 63 oil immersion objective. Images were collected at 10 second intervals. All images were processed by Volocity software (Improvision).

#### Statistical analysis

Statistical significance was tested using one-way analysis of variance (ANOVA) between groups and a series of  T-tests using GraphPad Prism version 3.02 (GraphPad Software, San Diego, California, U.S.A.), p < 0.05 was considered as significant.

### Results

#### Inhibition of GSK-3 slows T-cell motility

**Fig. 1:**
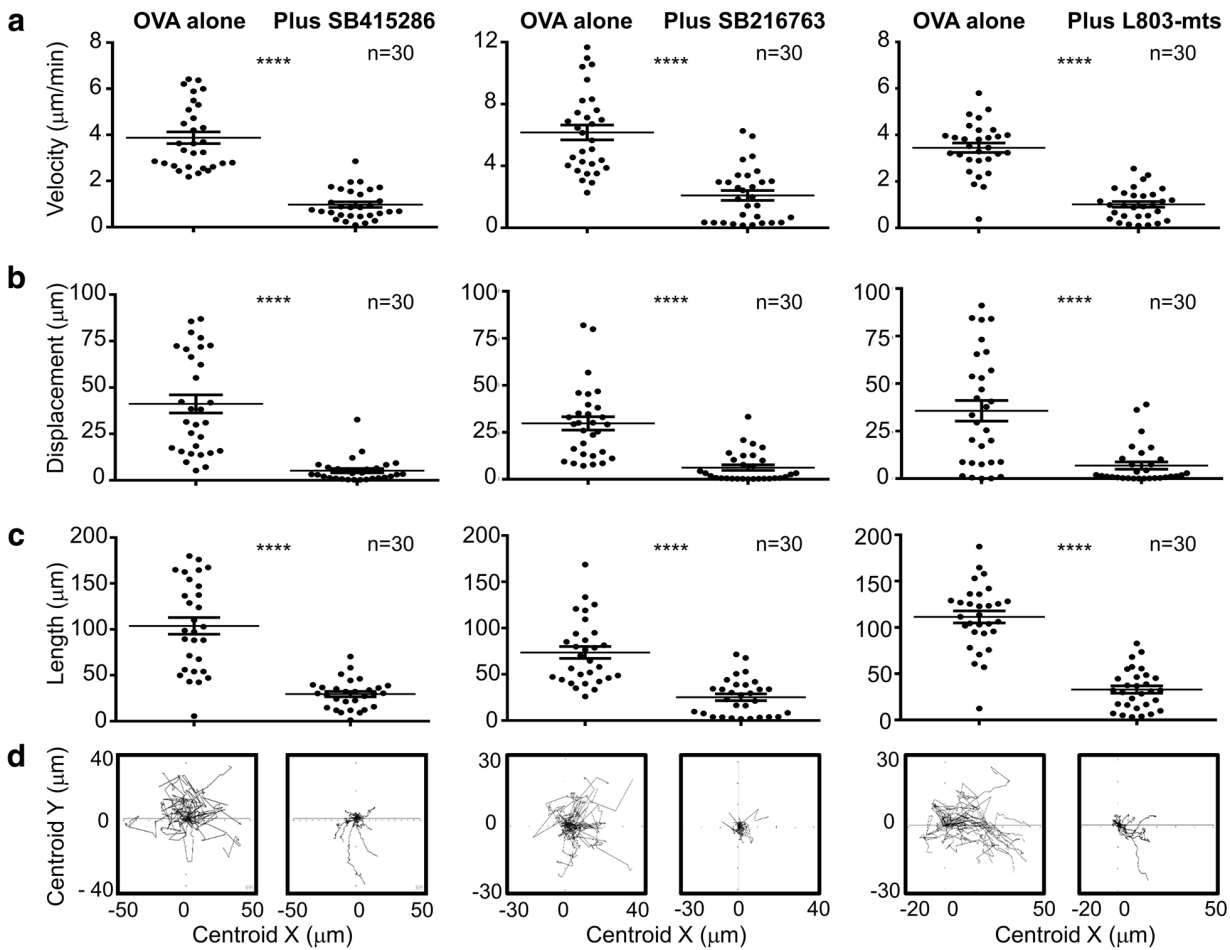
GSK-3 inhibition decreases T-cell motility. Cells treated with GSK-3 inhibitors (Left panel: SB415286, Middle panel: SB216763, right panel: L803-mts) for 7 days show reduced motility in the presence of target cells (EL4-OVA). Tracking of 30 individual cells showed differences in **a** velocity, **b** displacement and **c** length of track travelled. Spider plots (**d)** show the traced tracks of all cells in area imaged. Data shown representative of three independent experiments. **** P value < 0.0001. Mean shown ± standard deviation

**Fig. 2:**
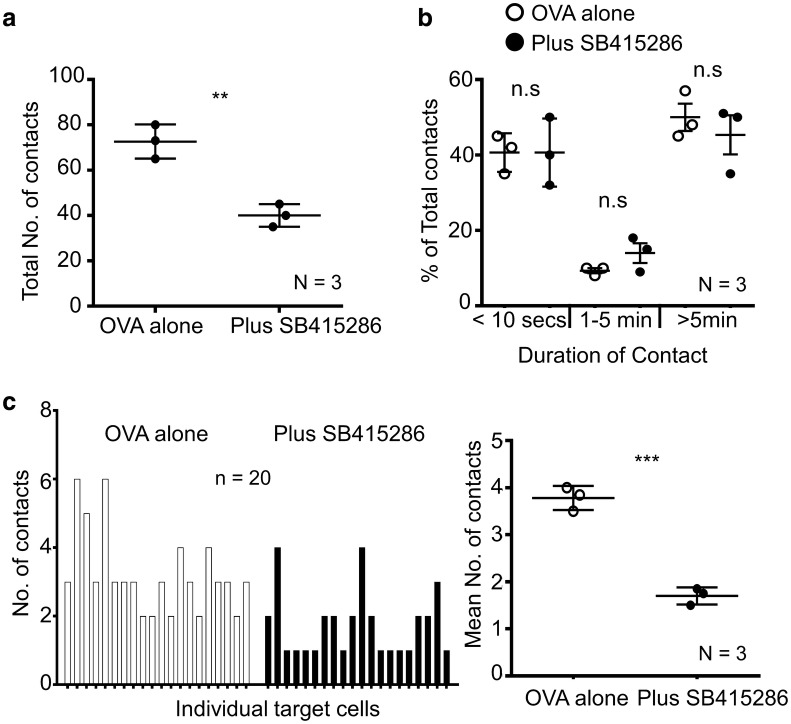
SB415286 decreases T-cell contacts with other cells. Inhibition of GSK-3 reduces the number of cell-to-cell contacts required to induce target killing. Quantification of contact times **a** total number of contacts for each condition, (Ova alone Mean = 72.67 ± 4.3, Plus SB415286 Mean = 40.00 ± 2.89). **b** GSK3 inhibition does not alter the dwell times of T-cells. % of contacts with different durations of contact is shown. Data shown is pooled from (N) = 3 independent experiments. *n.s* no significant difference. **c** Left panel, number of contacts by each individual target cell tracked (n = 20 Target cells (EL4-OVA)). Right panel, Mean number of contacts by individual target cells from (N) = 3 independent experiments (Ova alone Mean = 3.8 ± 0.15, Plus SB415286 Mean = 1.7 ± 0.10). * P < 0.05; ** P < 0.005; *** P < 0.0005

In order to assess the role of GSK-3 in T-cell motility, T-cells (OT-1 Tg) were initially imaged over a period of 5 min in the presence or absence of GSK-3 inhibitors (Fig. 1a), SB415286 (left panel), SB216763 (middle panel) and L803-mts (right panel). In the absence of SB415286, T-cells moved with a mean velocity of 4µm/min with a wide range of motilities from 7µm/min to 2µm/min. The presence of SB415286 slowed cells with an average velocity of 1um/min (i.e. 75% reduction). Similar profiles were observed when other parameters were used to assess movement (Fig. 1b, c). The displacement showed a reduction from 46µm to 2.5µm (Fig. 1b) and the length also was reduced from 104µm to 30µm (Fig. 1c). Spider graphs also illustrated the reduced distance travelled over time (Fig. 1d). Similar results were obtained using other structurally distinct inhibitors of GSK-3, all of which have been previously shown to decrease PD-1 expression and potentiate OT-I killing of targets [22, 23]. These included ATP-competitive inhibitors, L803-mts (Fig. 1, Right panels) and SB216763 (Fig. 1, middle panels). The peptide L803-mts (11 residues) is a cell-permeable phosphorylated peptide that is derived from the GSK-3 substrate heat shock factor-1 (HSF-1) and is structurally unrelated to SB415286 and SB216763 [25]. SB216763 has a preference for the GSK-3alpha isoform, while L803-mts preferentially inhibits GSK-3beta. These data therefore collectively showed that GSK-3 kinase activity is needed for the optimal migration of T-cells (Fig. 2).

The result of reduced motility could lead to an increase or decrease in contacts with other cell types. For example, disruption of adaptors needed for integrin binding reduces contacts with antigen presenting cells [26, 27]. Interestingly, the presence of SB415286 reduced the total number of contacts of OT-1 Tg T-cells with antigen-presenting cells by approx. 50% (Fig. 2a), with a mean of 73 contacts in the untreated cells verses 40 contacts in the inhibitor-treated cells (SD = 4.3 and 2.9, respectively); however, surprisingly, GSK-3 inhibition had no significant effect on the duration of cell contact which occurred (Fig. 2b). This reduction of contacts can be seen further when looking at individual target cells (Fig. 2c). Fig. 2c (left panel) shows 20 individual target cells (EL 4-OVA) and the number of contacts made. In the absence of SB415286 the mean number of contacts was 3.25 (SD ± 0.27). This was reduced to a mean of 1.75 contacts (SD ± 0.22) in the presence of SB415286. The right panel of Fig. 2c depicts the mean number of contacts from 3 independent experiments giving an overall mean of 3.8 ± 0.15 in the absence of SB415286 and 1.7 ± 0.1 in the presence of SB415286. These data show for the first time that GSK-3 activity is needed for optimal interactions of T-cells with other cells.

**Fig. 3:**
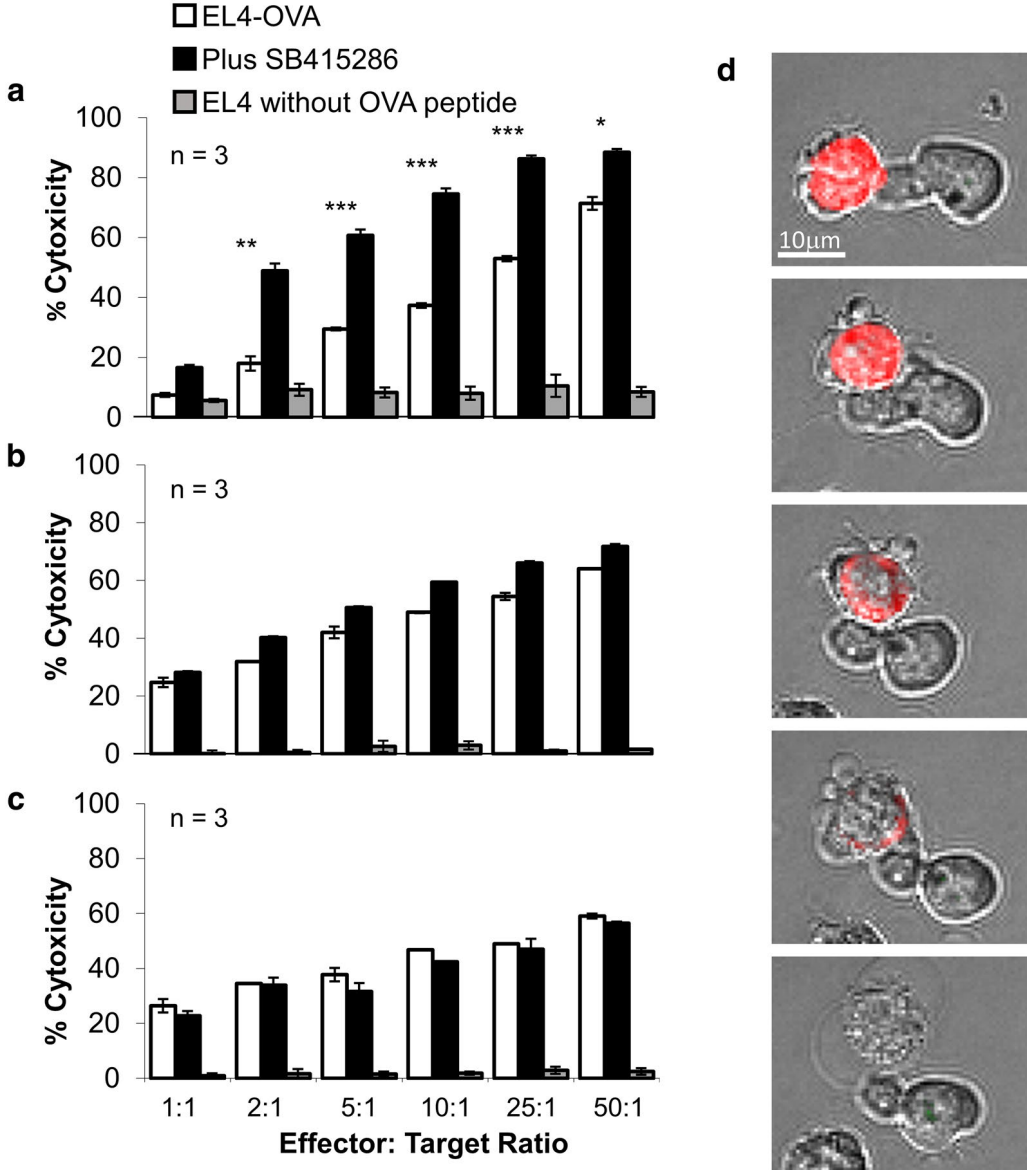
GSK-3 inactivation requires long-term incubation with T-cells to enhance CTLs killing of tumors. GSK inhibitor increases cytolytic killing of CTLs when present during over the cell culture period needed to induce differentiation. CTLS were generated by incubating splenocytes from OT-1 Tg mice with OVA-peptide for 7 days. SB415286 was added to cultures on **a** day 0 or **b** day 6. On day 7 T-cells were washed and a 4 h cytolytic assay performed using EL-4 cells pulsed or non-pulsed with Ova-peptide as target cells.  **c** depicts T-cells only treated with SB415286 following the wash step and prior to the 4 h CTL assay (In panels **a** and **b** any residual SB415296 was washed away). Error bars based on triplicate values in individual experiments, data shown representative of 3 independent experiments. * P < 0.05; ** P < 0.005; *** P < 0.0005. **d** Figure shows examples of T-cells interacting and killing tumor targets (EL4-OVA cells labelled in red). The CTL then goes on to lyse the cell as can be seen from the characteristic bubbling of the cell cytoplasm and the clear vacuole. Data shown representative of three independent experiments

To address whether the effects of GSK-3 inactivation on reducing T-cell motility and numbers of contracts was reflected in target killing, we next cultured CTLs with SB415286 for various times and assessed levels of killing (Fig. 3). We previously reported that long term exposure of primary T-cells to SB415286 increased the potency of killing by resultant CTLs [22–24]. Indeed, the culturing of T-cells for 7 days in the presence of SB415286 potentiated the killing of EL4-OVA targets. The increase in killing efficiency seen was 3- to 5-fold for effector:target ratios of 2:1 through to 25:1. An effector:target ratio of 2:1 with inhibitor showed the same efficiency of killing as seen at a ratio of 25:1 for non-inhibitor treated cells (Fig. 3a). By contrast, we next assessed whether the presence of GSK-3 inhibitors had an effect following the actual generation of armed CTLs, the generated CTLs were exposed to SB415286 for 24 h **(**Fig. 3b**)** or 4 h **(**Fig. 3c**)** and were assessed for killing. The brief exposure of CTLs to SB415286 had no detectable effect after a 4 h incubation, the same period that we showed could affect T-cell motility. Fig. 3d shows an example of the killing of a tumor target with the bubbling of membranes characteristic of cell death. Therefore, surprisingly, over the time frame where SB416286 could affect motility, no detectable effect on the killing of tumor targets was evident. The CTLs were, therefore, sufficiently potent in killing targets so that the GSK-3 effects on T-cell motility and numbers of contacts did not appreciably affect the overall killing of tumor targets in the in vitro assays of killing.

### Discussion

Overall, the rationale of our study was to assess whether GSK-3 inhibition effects are seen at the level of T-cell velocity and interactions with other cells. Our study shows that GSK-3 plays a clear role in regulating the movement of T-cells and interactions with other cells. Indeed, the inhibition of GSK-3 reduced the velocity of T-cells as measured in vitro on plates coated with ICAM1 for adhesion. The net result of this was to reduce T-cell velocity and also reduce the distance, otherwise needed to travel to interact with other cells. By contrast, the actual duration of the cell-cell interactions or the dwell times was not affected by GSK-3 inhibition. Movement is needed for T-cells to stochastically encounter other cell types or to respond to chemo-attractants such as chemokines.

The fact that GSK-3 inhibition does not have an effect within minutes of exposure but rather requires longer periods of incubation following activation suggests that its effect on motility may be less important for the effects of GSK-3 inhibition on CTL klling may be less important than the more long term effects of inhibition on T-cell activation or differentiation. Naive murine T-cells become effector T-cells followed by the generation of central memory T-cells [28]. We previously showed that GSK-3 regulates this event leading to more potent CTLs [22–24, 29, 30]. The potential disadvantage of reduced motility and interactions with other cells appears to be overridden by the positive intracellular effects on CTL differentiation [22–24, 29].

Further, it is important to note that different inhibitors of GSK-3 had the same effect on T-cell motility. L803-mts is structurally unrelated to SB415286 and SB2167763 [25]. Further, SB216763 has a preference for the GSK-3alpha isoform, while L803-mts preferentially inhibits the GSK-3beta isoforms. Despite different structures and isoform specificities, the exposure of cells to all drugs overtime resulted in population of cells with reduced motility after long-term exposure.

The underlying mechanism of GSK-3 on T-cell motility is not clear. As mentioned, the effects require long-term incubation with T-cells, and are, therefore, most likely related to effects on the activation or differentiation of T-cells. However, effects on more proximal events are also possible since GSK-3 can phosphorylate microtubule-associated protein 2C (MAP2C) which prevents microtubule remodelling [16, 17]. It is also possible that GSK-3 interfaces with adaptor proteins such as SKAP1 which regulate T-cell motility [31]. The N-terminal region of SKAP1 binds to RapL such that a RapL mutation (L224A) abrogates SKAP1 binding and arrests T-cells even in the absence of antigen [17]. Lastly, it is possible that GSK-3 may influence cell motility and chemotaxis by regulating Phosphoinositide 3-kinase (PI 3 K) membrane localization as has been observed in *Dictyostelium* [32] or due to effects on phosphatidylinositol-3,4,5-triphosphate (PIP3) metabolism, the target of rapamycin complex (TORC)2 signaling, and remodeling of F-actin [33, 34]. Teo et al. have reported that *gsk3*^−^ cells respond to stimuli with a reduced increase of PIP3 and no TORC2 activation [34], decreased adenylyl cyclase, while others have obtained different results [33]. Future work will be needed to assess the full range of effects of GSK-3 on aspects of T-cell function linked to motility and migration.

## Limitations

Work restricted to non-lymphoid cells.

## Data Availability

All relevant material will be freely available to any scientist wishing to use them for non-commercial purposes. Data related to the tables, graph and calculation are available from the corresponding author upon request.

## Abbreviations

SLP-76SH2: SH2-domain containing leukocyte protein of 76 kDa
ADAP: Adhesion and degranulation-promoting adapter protein
GSK-3: Glycogen synthase kinase-3
LAT: Linker for activation of T-cells
SKAP1 (aka SKAP55): Src kinase-associated phosphoprotein-1
PIP3: Phosphatidylinositol-3,4,5-triphosphate
MAP2C: Microtubule-associated protein 2C
HSF-1: Heat shock factor-1
